# A Randomised Controlled Trial of IPS in Severe Mental Disorders: Mental Health, Functional, and Vocational Outcomes in a High-Unemployment Setting

**DOI:** 10.3389/ijph.2025.1608796

**Published:** 2025-12-08

**Authors:** Francisco Rodríguez Pulido, Dácil Oramas Pérez, Guadalberto Hernández Hernández, Enrique González Dávila, Nayra Caballero-Estebaranz

**Affiliations:** 1 University of La Laguna, Santa Cruz de Tenerife, Spain; 2 Canarian Health Service, Santa Cruz De Tenerife, Spain; 3 European University, Canary Islands Health Research Institute Foundation (FIISC), Santa Cruz de Tenerife, Spain; 4 Sinpromi, Santa Cruz De Tenerife, Spain

**Keywords:** schizophrenia, supported employment, rehabilitation, psychosocial functioning, quality of life

## Abstract

**Objective:**

This randomised controlled trial examined whether individuals with severe mental disorders (SMD) in a high-unemployment context benefit in mental health, functional and vocational outcomes when receiving the Individual Placement and Support (IPS) model, compared to vocational rehabilitation (VR).

**Methods:**

A total of 557 individuals (ICD-10 F20–F29 or F31–F32.3) were screened. 63 met inclusion criteria and voluntarily participated. They were randomly assigned to IPS or VR groups. Assessments were conducted at baseline and six months. The study was ethically approved, registered and conducted with blinded evaluations.

**Results:**

IPS participants achieved significantly higher rates of competitive employment and showed greater improvements in global functioning compared with VR. Other mental health and functional outcomes showed no significant group differences. However, participants who obtained employment reported greater improvements in quality of life. No adverse events, such as hospitalizations, were reported.

**Conclusion:**

These findings suggest that IPS may be effective beyond employment outcomes, with some benefits in mental health and functional domains. Despite limitations such as short follow-up and modest sample size, the study supports the feasibility of IPS in challenging labor markets.

## Introduction

About 8 in 10 people with mental health problems are unemployed (World Health Organization (WHO), 2022) and about 60%–70% of people with severe mental disorders (SMD) prefer paid employment [1, 2], over participation in sheltered workshops [3] or day centers [4].

Despite the emotional and psychological stressors associated with work, which are often unavoidable, unemployment is generally more detrimental than employment. Mental health problems [5] linked to unemployment include increased cognitive impairment, emergence of psychotic symptoms, fear of losing benefits [6–8], higher risk of substance abuse [9], and even self-harm ideation. Unemployment is associated with a 20%–30% relative increase in suicide risk [10].

The social, economic and psychological value of employment contributes to decreased symptoms and increased likelihood of recovery [11–15].

In mental health, various strategies have historically been used to address barriers to employment. Employment outcomes have also become a growing focus of scientific literature in recent years [16]. The high unemployment rates among people with schizophrenia have led to systematically evaluated interventions. One of these strategies is Individual Placement and Support (IPS), developed in the United States by Becker and Drake [17]. IPS emphasizes rapid entry into competitive employment according to individual preferences, integration of mental health and employment specialists, followed by ongoing supports and zero exclusion. Individuals with SMD are integrated into the workplace under the same conditions as those without mental disorders, thereby promoting social inclusion, reinforcing citizenship, and making work an essential component of the recovery process [18].

There is strong evidence from both Europe and the United States that the IPS model is more effective than vocational rehabilitation programs, such as “Train-then-Place”, for helping people with SMD obtain competitive employment [19]. Moreover, IPS is associated with better vocational outcomes, including more hours and weeks worked competitively and fewer hospital admissions [20]. While the literature on the effect of IPS on mental health and functional outcomes is less extensive, the European EQOLISE trial [21], a multicenter study, is the most representative of this effort in Europe and served as a reference for the present study, conducted in the context of high unemployment in the general population.

The purpose of this study is to investigate whether, in a high unemployment context such as the Canarian population, people with SMD benefit in their mental health, functional and vocational outcomes to obtain and maintain competitive employment with IPS versus vocational rehabilitation.

## Methods

This was an open-label, community-based, multicenter, randomized controlled experimental trial evaluating the effectiveness of Individual Placement and Support (IPS) versus vocational rehabilitation (VR, train-then-place) for achieving competitive employment in individuals with severe mental disorders (SMD), following the guidelines of the work of McGurk, Mueser and Pascaris [22].

### Sample

The participants in this study were recruited between 2021 and 2023 from the Community Mental Health Teams (CMHT) and basic health areas of Tenerife (Spain), one of the eight Canary Islands, geographically located in southern Europe. In 2021, Tenerife had a resident population of 930,570, and the Canary Islands reported a GDP *per capita* of €19,021, with the service sector as the predominant economic activity [23]. The unemployment rate in the Canary Islands has traditionally been among the highest in Spain, averaging around 20% during the study period. At the onset of the study (2021), unemployment exceeded 25%, gradually decreasing to about 14% by 2023. These figures remain substantially higher than the national average in Spain (approximately 14% in this period) and, than those reported in the European EQOLISE trial (around 10%–12%), thus supporting the characterization of this setting as a high-unemployment context.

The sample size calculation was based on the total score on the Positive and Negative Syndrome Scale (PANSS) as the primary outcome variable. A clinically relevant difference of 8.3 points between groups was assumed, with a standard deviation of 9.9 points, based on Leddy-Stacy and Rosenheck [24]. Using a 95% confidence level and 80% statistical power, the required sample size was 24 patients per group. To account for potential attrition, this number was increased by 30%, resulting in an initial target of 32 patients per group (31 in the IPS group due to one last-minute withdrawal).

**FIGURE 1:**
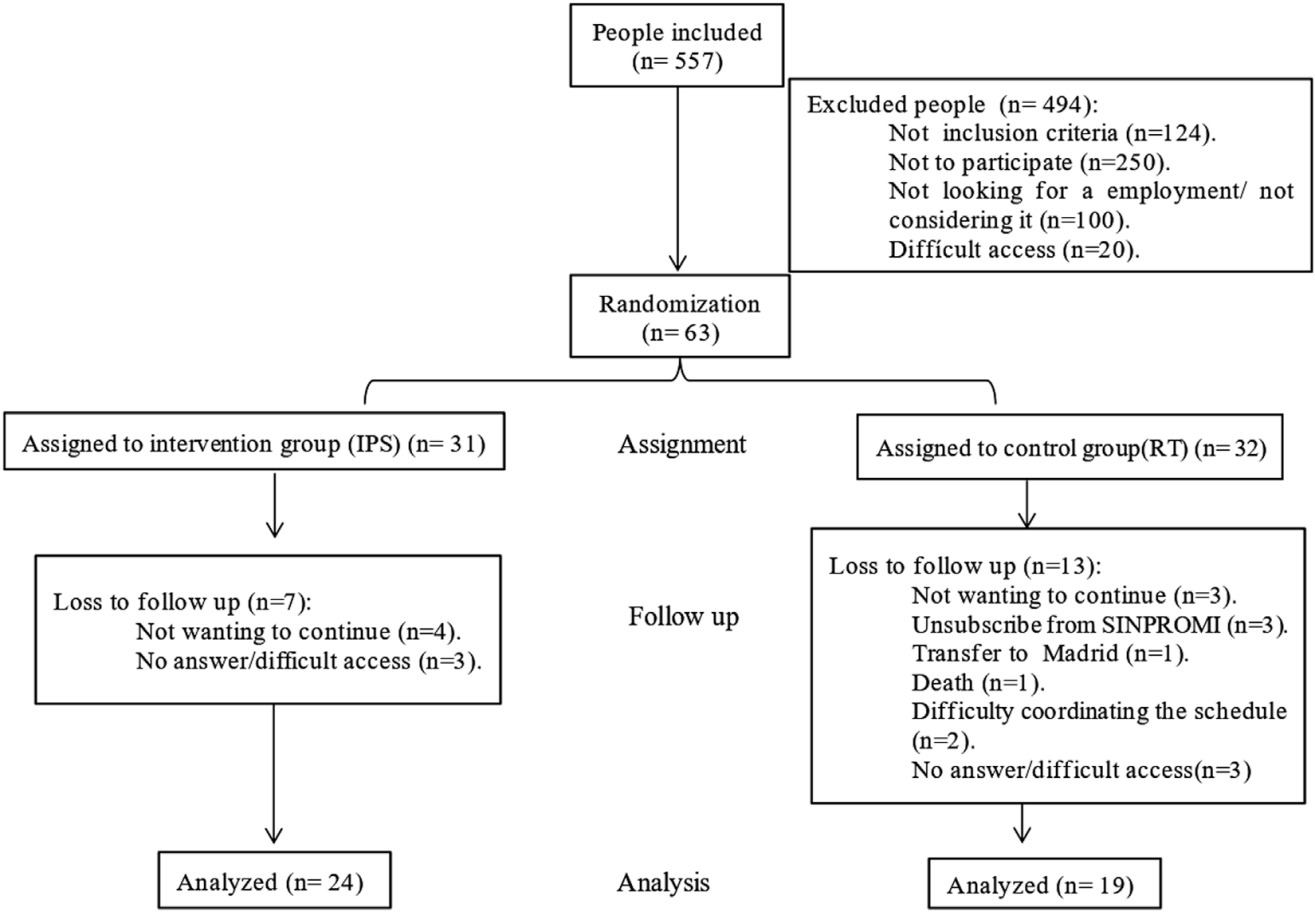
Flow diagram (Canary, Spain. 2024).

A total of 557 participants were recruited, of whom 494 were excluded for different reasons (see flowchart, Figure 1). The high number of participants who declined participation (n = 250), was primarily related to the lingering effects of the COVID-19 context, which limited willingness to engage in face-to-face activities, along with other factors such as concerns about stigma, self-perceived inability to work, low motivation to engage in employment programs, and practical barriers (e.g., limited time availability, transportation difficulties, and caregiving responsibilities). The remaining 63 eligible participants were randomly and blindly assigned to either IPS group (n = 31) or the VR group (n = 32). During the follow-up, 7 participants in the IPS group and 13 in the VR group discontinued participation, leaving 24 and 19 participants, respectively, for the final analysis. All the participants gave their verbal and written informed consent to participate in the study.

### Inclusion and Exclusion Criteria

The inclusion criteria used were the following: age between 18 and 65 years, diagnosis of schizophrenia (ICD-10 diagnoses), capacity to consent to voluntary participation in the study, being clinically stable without acute psychotic symptoms or having been hospitalized during the last months, being followed by the public community mental health teams during the study and being motivated to obtain a regular job.

The exclusion criteria used were having a common mental and/or other organic disorder, having a history of head injury with loss of consciousness, and being pregnant or breastfeeding.

The criteria foreseen for the withdrawal of subjects from the study were the explicit request by the subject to stop participating in the study, difficulty traveling to the place of the interviews, scheduling conflicts, change of place of residence outside the autonomous community where the study was carried out or death.

### IPS Program and Fidelity

IPS was first implemented in Tenerife in 2004, following the scientific literature of the Dartmouth IPS Center. Since then, the program has been coordinated by the Insular Council of Psychosocial Rehabilitation and Community Action of the Canary Health Service together with Simpromi S.L., in collaboration with the University of La Laguna. The program has stable public funding, integration with mental health services, and has been described in previous publications [25, 26]. Its consolidation was further strengthened by hosting the first IPS Spain meeting, which paved the way for its subsequent inclusion in the IPS International Learning Community (IPS Europe, 2023) [27].

Fidelity to the IPS model was ensured by consistently adhering to the core IPS principles (zero exclusion, focus on competitive employment, integration with clinical services, rapid job searches, individualized support). As reported in [26], a fidelity assessment conducted in 2015 using the Supported Employment Fidelity Scale (IPS-15) yielded a score of 66 out of 75, corresponding to good implementation. In addition, a more recent fidelity assessment was conducted in 2020, prior to the current study period, using the Supported Employment Fidelity Scale by Becker et al. (IPS-25) [28]. This evaluation, performed by an independent psychologist not affiliated with the IPS team, yielded a score of 106 out of 125, consistent with good fidelity to the IPS model (unpublished data).

### Measuring Instruments

The principal investigator (PI), who was blinded to group assignment, assessed mental health, clinical and functional outcomes at baseline and at six-month follow-up. The following instruments were used:

Global Assessment of Functioning Scale (GAF) [29]. GAF is a numerical and descriptive scale that provides a single score reflecting a patient’s overall psychological, social, and occupational functioning. Scores range from 0 to 100, with higher scores indicating better functioning.

Lancashire Quality of Life Test and Satisfaction with Life Scale (ESV) [30]. This is a structured interview to measure the health and wellbeing of individuals with mental disorders. Subjective ratings are made using a visual analog scale (Life Satisfaction Scale) (1 = worst, 7 = best). The instrument also assesses global wellbeing and the interviewer scores the patient’s quality of life and makes an estimate of the reliability of the responses.

Positive and Negative Syndrome Scale (PANSS) [31]. It assesses symptomatology. The PANSS is an interview and observation-based scale consisting of 30 items: 7 for the positive scale (PANSS-P), 7 for the negative scale (PANSS-N) and 16 for the general psychopathology scale (PANSS-PG). Each item is evaluated according to a Likert scale of 7 degrees of intensity, where 1 is always equivalent to the absence of symptoms and 7 represents extreme severity.

Anxiety-Depression Scale (HADS) [32]. It is a self-administered questionnaire of 14 items, composed of two subscales of 7: one for anxiety (odd items) and another for depression (even items). The intensity or frequency of the symptom over the previous week is evaluated on a 4-point Likert scale (range 0–3).

Groningen Social Disabilities Schedule Test (GSDS) [33]. Based on social role theory, the GSDS evaluates social functioning across multiple domains, including self-care, family relationships, marriage, parenthood, citizenship, social, and occupational roles.

AF5 self-concept test [34]. It consists of 30 items, equally divided into 5 dimensions of self-concept: academic/work, social, family, emotional and physical. Responses are recorded on a scale from 1 (strongly disagree) to 99 (strongly agree).

In addition to the variables obtained from the aforementioned instruments, relevant clinical history data were collected from participants, including hospitalizations (number of admissions to short- or medium-stay beds during the past 6 months, as determined by the treating psychiatrist) and relapses (defined as exacerbations of symptoms measured by the PANSS).

### Procedure

The study was approved by the ethics committee (CI 2024-669) and conducted in accordance with the Declaration of Helsinki and European/Spanish legislation. Written informed consent was obtained from all participants, ensuring confidentiality and data protection.

Participants were referred from public CMHTs to IPS teams, which consisted of employment specialists from SINPROMI (Sociedad Insular para la Promoción con Discapacidad). The principal investigator (PI) was independent from these services and blinded to group assignment during recruitment and baseline assessments. Randomization occurred only after informed consent and baseline evaluation, minimizing selection bias.

Initial interviews collected sociodemographic level, educational level, previous work history and comorbidities. Participants were informed that employment could be obtained at any time, either during IPS or following VR. In addition, previous employment information, income and hospitalizations prior to the study were collected. Validated scales for mental health and functional outcomes were administered at baseline and repeated at six-month follow-up, together with employment and hospitalization data.

### Statistical Analysis of Data

Data from the surveys were entered into an Excel spreadsheet and statistical analysis was performed using the Statistical Package for the Social Sciences (SPSS) Program (version 25, 2017) and GraphPad Prism 9.

To describe the qualitative variables included, the count and percentage were calculated, while the mean and standard deviation were used for the continuous variables. During the baseline phase, the comparison of the continuous variables was performed with the t-student test for independent samples, after verifying that they followed a normal distribution (Kolmogorov-Smirnov test). The chi-square test was used to compare qualitative variables, and in the case of 2 × 2 tables in which the expected frequency was less than 5, Fisher’s exact test was used.

The comparison of the information obtained in the baseline and follow-up phases for the quantitative variables of the study was carried out with a repeated measures analysis of variance (RM-ANOVA). Group and the interaction between group and time (baseline and follow-up) were introduced as a factor. In addition to statistical significance, effect sizes (Cohen’s d) were calculated to quantify the magnitude of within-group and between-group changes over time.

Cohen’s Kappa coefficient of concordance was used to analyze the evolution of the categorical variables between baseline and 6 months for the GSDS scale. In those cases, in which only two categories (“excellent” and “satisfactory”) were given, McNemar’s test was additionally calculated.

A multivariate logistic regression model was used to analyze employability data, with explanatory variables selected using the Backward Wald method.

All statistical tests were two-tailed, with a significance threshold of p < 0.05. Given the exploratory nature of this trial and the modest sample size, no correction for multiple comparisons was applied. Findings should therefore be interpreted with caution and considered preliminary.

## Results

A total of 63 participants were recruited from a group of 557. At the 6-month follow-up, 43 remained in the study (24 IPS and 19 VR), with 20 participants lost for reasons detailed in Figure 1. No significant differences in sociodemographic characteristics were observed between groups at baseline (Table 1), supporting sample homogeneity. The majority were male, aged 36–45 years, with an educational level (43%) in vocational training, and most had a diagnosis of schizophrenia or bipolar disorder.

**TABLE 1:** Baseline characteristics of the sample and comparison of participants of completers vs. dropouts by groups, n (%) (Canary, Spain. 2024).

Variable	Baseline sample				VR group			IPS group		
	VR (n = 32)	IPS (n = 31)	Total (n = 63)	p-value	Completers (n = 19)	Dropout (n = 13)	p-value	Completers (n = 24)	Dropout (n = 7)	p-value
Sex				0.250			1.000			0.191
Man	23 (72)	18 (58)	41 (65.1)		14 (73.7)	9 (69.2)		12 (50)	6 (85.7)	
Woman	9 (28)	13 (42)	22 (34.9)		5 (26.3)	4 (30.8)		12 (50)	1 (14.3)	
Age groups (years)				0.186			0.310			0.856
18–25	-	3 (10)	3 (4.8)		-	0		3 (12.4)	0	
26–35	11 (34)	7 (23)	18 (28.6)		6 (31.6)	5 (38.5)		6 (25)	1 (14.3)	
36–45	12 (38)	15 (48)	27 (42.9)		7 (36.8)	5 (38.5)		11 (45.8)	4 (57.1)	
46–55	9 (28)	6 (19)	15 (23.8)		6 (31.6)	3 (23.0)		4 (16.7)	2 (28.6)	
Previous work experience	29 (91)	30 (97)	59 (93.7)	0.317	17 (89.5)	12 (92.3)	1.000	23 (95.8)	7 (100)	1.000
Level of education				0.160			0.465			0.819
Primary	9 (28)	12 (39)	21 (33.3)		7 (36.8)	2 (23.2)		9 (37.5)	3 (42.9)	
Secondary	7 (22)	1 (3)	8 (12.7)		3 (15.8)	4 (30.8)		1 (4.2)	0	
Baccalaureate	3 (9)	1 (3)	4 (6.4)		1 (5.3)	2 (15.5)		1 (4.2)	0	
FP (vocational training)	12 (38)	15 (48)	27 (42.9)		7 (36.8)	5 (38.5)		12 (50)	3 (42.9)	
University	1 (3)	2 (7)	3 (4.8)		1 (5.3)	-		1 (4.2)	1 (14.2)	
Hospitalizations in the last 6 months prior to the start of the study	-	1 (3)	1 (1.6)	0.492	0	0	1.000	1 (4.2)	0	1.000
No medical problems	14 (44)	15 (48)	29 (43.0)	0.712						
Diagnosis				0.556			0.538			0.078
Schizophrenia	20 (62)	13 (42)	33 (52.4)		10 (52.6)	10 (79.9)		9 (37.5)	4 (57.1)	
Bipolar disorder	5 (16)	6 (19)	11 (17.5)		4 (21.1)	1 (7.7)		7 (29.2)	0	
Personality disorder	2 (6)	3 (10)	5 (7.9)		1 (5.3)	1 (7.7)		1 (4.2)	2 (28.6)	
Recurrent depressive disorder	-	1 (3)	1 (1.6)		0	0		1 (4.2)	0	
Disorder delusional ideas	2 (6)	2 (6)	4 (6.4)		2 (10.5)	0		0	1 (14.3)	
Schizoaffective disorder	3 (9)	4 (13)	7 (11.1)		2 (10.5)	1 (7.7)		4 (16.7)	0	
Others	-	2 (6)	2 (3.2)		0	0		2 (8.3)	0	

VR, Vocational rehabilitation (train-then-place); IPS, Individual Placement and Support.

The sociodemographic characteristics of participants who dropped out (see Table 1) revealed no significant differences compared with those who completed the study, suggesting that attrition was not related to baseline characteristics, including diagnosis type.

### Mental Health and Functional Outcomes

Mental health and functional outcomes are summarized in Supplementary File 1. Overall, IPS participants showed greater improvements in global functioning (GAF) compared with VR, while other non-vocational outcomes-quality of life, symptomatology (PANNS), anxiety and depression (HADS), social functioning (GSDS), and self-concept (AF)- did not differ significantly between groups. Improvements over time were noted in both groups, particularly in GAF and quality of life, suggesting that participation in either program may have beneficial effects. The only non-vocational outcome showing a group effect was GAF, with significantly better scores in the IPS group, consistent with its inclusion of functional domains related to employment.

As shown in Table 2 and Supplementary File 2, repeated-measures ANOVA indicated that, among participants who completed follow-up, PANSS scores decreased in both groups at 6 months, reflecting an overall reduction in psychotic symptoms, anxiety, and depression. A significant time × group interaction was observed for social self-concept (p = 0.014), participants in the IPS group improved over time, whereas those in the VR group showed a decline. For all other non-vocational variables assessed (quality of life, other self-concept dimensions, and social functioning), no significant group differences were observed, indicating that improvements over time were similar in both IPS and VR participants. Effect sizes (Cohen’s d) for within-group and between-group changes over time are presented in Supplementary File 3. Consistent with the inferential results, medium to large effect sizes were observed for improvements in overall functioning (GAF) and in the social self-concept domain, favoring the IPS group. Within-group analyses also showed large effects for reductions in psychotic symptoms across both groups.

**TABLE 2:** Evaluation of Individual Placement and Support (IPS)/Vocational Rehabilitation (VR) strategies on evolution of mental health and functional outcomes (means and standard deviations), using repeated measures analysis of variance (RM-ANOVA) (Canary, Spain, 2024).

		Control group (VR) (n = 19)		Intervention group (IPS) (n = 24)		p -value		
		Basal	6 months	Basal	6 months	Time	Group	Time x group
Quality of life		4.8 (0.9)	5.0 (1.0)	5.2 (0.8)	5.5 (0.8)	0.034	0.108	0.438
GAF		57.74 (8.32)	59.2 (8.3)	63.8 (10.8)	68.0 (9.0)	0.002	0.009	0.114
PANSS	Positive	15.16 (5.1)	10.8 (3.7)	14.9 (7.3)	11.9 (5.1)	<0.001	0.804	0.308
	Negative	18.7 (6.4)	13.83 (5.2)	19.4 (7.9)	13.2 (5.7)	<0.001	0.704	0.985
	General	37.8 (11.1)	29.4 (9.1)	36.0 (12.3)	28.7 (8.3)	<0.001	0.659	0.740
HADS	Anxiety	5.0 (3.7)	4.2 (2.8)	4.5 (3.7)	4.1 (2.7)	0.244	0.704	0.676
	Depression	4.3 (3.4)	4.1 (4.0)	4.3 (3.5)	3.5 (3.6)	0.231	0.793	0.485
	Global	9.3 (6.4)	8.3 (6.3)	8.8 (6.6)	7.63 (5.8)	0.176	0.731	0.916
Self-concept	Academic	7.1 (1.3)	6.7 (1.0)	7.4 (1.8)	7 (1.54)	0.042	0.460	0.911
	Social	6.5 (1.9)	5.8 (1.5)	6.5 (2.1)	6.7 (2)	0.287	0.403	0.014
	Emotional	5.9 (2.4)	6.0 (1.6)	6.1 (2.5)	5.8 (1.63)	0.673	0.961	0.612
	Family	7.6 (1.8)	7.8 (1.7)	7.4 (2.2)	7.4 (2.1)	0.606	0.589	0.064
	Physicist	5.3 (2.6)	4.7 (1.8)	5.6 (2.5)	5.4 (2.47)	0.108	0.486	0.268
GSDS total		10.3 (4.0)	9.9 (3.7)	9.2 (4.6)	9.3 (4.6)	0.647	0.501	0.400

The relationship between baseline and six-month scores on each scale showed higher Pearson correlation coefficients in the IPS group compared with VR (GAF: 0.855 vs. 0.772; PANSS positive: 0.897 vs. 0.518; PANSS negative: 0.658 vs. 0.488; PANSS global: 0.761 vs. 0.668; HADS global: 0.798 vs. 0.603, respectively). These findings suggest a stronger association between initial functioning and subsequent improvement among participants receiving IPS.

High concordance values (close to 1) were observed between baseline and six-month evaluations, indicating that employment interventions did not produce changes or worsen patients’ conditions, as reflected in GSDS domain scores. No significant differences were found between the groups.

When comparing completers with dropouts (Supplementary File 4), participants who discontinued the study had lower scores on the PANSS negative and general subscales, as well as on HADS depression. This pattern suggests that individuals with fewer symptoms were more likely to drop out, whereas those with more severe symptomatology tended to remain in the study.

### Vocational Outcomes

Employment rates were markedly higher in the IPS group, where 18 of 24 participants (75.0%) obtained competitive employment, compared with 3 of 19 participants (15.8%) in the VR group (χ^2^ = 14.880, p < 0.001; Supplementary File 5). Logistic regression indicated that IPS participants were 16 times (95%CI 3.43, 74.70; p < 0.001) more likely to obtain competitive employment than those in VR.

To further explore whether employment status influenced mental health and functional outcomes, repeated-measures ANOVA was conducted (see Table 3). Being employed was associated with greater improvements in global functioning (GAF) and quality of life over time. Specifically, quality of life increased across both employed and unemployed participants (p = 0.026), but those who obtained employment experienced a significantly greater improvement (p = 0.043). Similarly, GAF showed significant changes over time (p = 0.001) and between groups (p = 0.001), with the largest gains among those who achieved employment.

**TABLE 3:** Evaluation of Individual Placement and Support (IPS)/RT strategies on mental health and functional outcomes at 6-month follow-up (means and standard deviations) applying repeated measures analysis of variance (RM-ANOVA) (Canary, Spain, 2024).

		Employment (n = 21)		Non-employment (n = 22)		p -value		
		Baseline	6 months	Baseline	6 months	Time	Group	Time × group
Quality of life		5.29 (0.78)	5.62 (0.74)	4.86 (0.94)	5.05 (0.95)	0.026	0.043	0.501
GAF		65.62 (10.89)	69.10 (8.84)	56.86 (7.25)	59.41 (8.05)	0.001	0.001	0.593
PANSS	Positive	14.7 (5)	11.1 (4.3)	15.3 (7.5)	11.7 (4.9)	<0.001	0.718	0.316
	Negative	19.7 (7)	13.9 (4.5)	18.5 (7.6)	13.2 (6.3)	<0.001	0.601	0.801
	General	36.4 (9.9)	29.2 (7.2)	37.2 (13.4)	28.8 (9.9)	<0.001	0.933	0.682
HADS	Anxiety	4.57 (3.37)	4.19 (2.66)	4.82 (4.02)	4.09 (2.84)	0.265	0.933	0.726
	Depression	4.62 (3.83)	3.62 (3.83)	4.05 (3.51)	3.95 (4.12)	0.186	0.908	0.269
	Global	9.2 (6.1)	7.81 (5.4)	8.9 (6.9)	8.05 (6.6)	0.167	0.979	0.720
Self-concept	Academic	7.5 (1.5)	7.20 (1.3)	7 (1.7)	6.6 (1.33)	0.042	0.15	0.885
	Social	6.5 (2.1)	6.5 (1.9)	6.4 (1.9)	6.1 (1.9)	0.401	0.632	0.511
	Emotional	5.9 (2.3)	5.6 (1.5)	6.2 (2.6)	6.1 (1.7)	0.623	0.444	0.751
	Family	7.6 (2.4)	7.6 (2.1)	7.4 (1.6)	7.5 (1.7)	0.636	0.795	0.851
	Physicist	5.3 (2.3)	5.2 (2.4)	5.7 (2.7)	5.1 (2.1)	0.143	0.847	0.316
GSDS total		8.2 (4.9)	8.2 (4.8)	11.1 (3.4)	10.8 (3.2)	0.725	0.033	0.725

For symptomatology, reductions were observed in both employed and unemployed participants across the three PANSS subscales (positive, negative, and general) over time (all p < 0.001). However, no significant group differences were found between those who obtained employment and those who did not (p = 0.718, p = 0.601, p = 0.933, respectively), indicating that employment status did not lead to symptom exacerbation or hospitalizations.

## Discussion

Work integration strategies are essential for the recovery of people with Severe Mental Disorders (SMD). Employment enhances identity, autonomy, and participation, while a rights-based perspective highlights the need to reduce structural barriers so individuals can make decisions on equal terms. Although 60%–70% of people with SMD want to work, 80%–90% remain unemployed [1, 35], not due to lack of motivation but because of fears of losing benefits, low self-confidence, limited support from health services [36, 37], stigma, institutional inertia, and underfunded policies.

This randomized trial confirmed the efficacy of the Individual Placement and Support (IPS) model in a high-unemployment setting. IPS participants achieved significantly higher rates of competitive employment (75% vs. 16% in VR), consistent with international evidence. This aligns with findings by Drake et al. [38], where IPS outcomes in Europe were 44% compared with 20% in traditional programs, and even higher in the United States and Australia. Brinchmann et al. [39] further confirms IPS’s broad applicability.

The high employability achieved in this study may be linked to the strong collaboration between public health services, employment specialists from SIMPROMI, and institutions promoting disability inclusion. The IPS program in Tenerife has stable public funding and is integrated into mental health services, features that have likely contributed to its success.

Regarding mental health and functional outcomes, IPS participants showed greater improvements in global functioning (GAF) and social self-concept compared with VR, while other domains (symptoms, anxiety, depression and remaining self-concept dimensions) improved similarly in both groups over 6 months. GAF includes consideration of functioning across multiple domains, including employment, which may account for the improvement observed. Importantly, participants who obtained competitive employment—regardless of intervention group—reported greater improvements in quality of life. Results were consistent with Wallström et al [40]. Kukla and Bond [41], in their randomised controlled trial, concluded that participation in supported employment alone is not sufficient to positively impact most non-vocational outcomes in people with SMD.

Measuring non-vocational outcomes remains challenging due to inconsistent metrics across studies. We adopted the EQOLISE trial as a Ref. [21], although results often vary depending on local economic conditions [42]. In our study, self-concept was incorporated as a key variable, differentiated from self-esteem. Negative self-perception, frequently observed in SMD due to stigma, can significantly affect motivation and social integration [43]. While Burns et al. [21] reported no clinical differences between IPS and vocational service recipients. Frederick and VanderWeele [44] in a randomised controlled trials also reported significant effects of IPS on non-vocational outcomes.

Employment was also associated with greater subjective wellbeing [11, 45, 46]. Within 6 months of obtaining work, participants in our study reported better social functioning, activity levels and quality of life. Furthermore, employment was linked to reductions in symptomatology, with PANSS scores showing improvement and no increase in hospitalizations. These results are consistent with findings by Mueser et al. [47] and by McGurk and Mueser [48], who emphasized the therapeutic value of employment in reducing negative symptoms and enhancing functioning. Previous research [21, 49] also shows that employed individuals have better clinical stability and fewer hospitalizations. Quality of life and self-esteem increase with competitive employment [50].

Employment stability is crucial. Consistent work for over 90 days correlates with fewer symptoms and improved functioning [21]. In our study, improvements in GAF, PANSS, and self-concept occurred over time in both groups, but were greater in those employed. The IPS model fosters sustained benefits, reinforcing the need for long-term follow-up strategies. Drake et al [51] show that competitive employment over extended periods stabilizes the beneficial effect of IPS on mental health and functional outcomes.

Several limitations should be noted. First, the relatively small sample size reduced statistical power, especially for secondary outcomes. Second, multiple outcomes were analyzed without strict correction for multiple comparisons, which increases the risk of type I error. Third, the follow-up period was limited to 6 months, providing only a short-term perspective, while employment stability and long-term effects remain unknown. Fourth, the dropout rate of 31.7% may have introduced bias, although no major baseline differences were observed between completers and non-completers. Finally, the specific socio-institutional context of Tenerife, with strong coordination across services, may limit generalizability to other regions. Additionally, the temporary nature of jobs raises concerns about future job stability, warranting further study. Furthermore, qualitative research, such as interviews or focus groups, could offer insights into users’ personal experiences and perceived benefits.

Conclusion: IPS is feasible and highly effective in increasing competitive employment for people with SMD, even in a high-unemployment context. Employment itself is beneficial for people with SMD, enhancing clinical and social functioning, self-concept and quality of life, without worsening symptoms or increasing relapse risk. Policies should prioritize personalized support and sustained funding to scale up IPS implementation. Future multicenter studies with larger samples, longer follow-up, and standardized evaluation protocols are needed to better capture mental health and functional outcomes and confirm the mediating role of employment in clinical and social recovery.
