# Supplementary material for: A Randomised Controlled Trial of IPS in Severe Mental Disorders: Mental Health, Functional, and Vocational Outcomes in a High-Unemployment Setting
Source: Int J Public Health. 2025 Dec 8;70:1608796. doi: 10.3389/ijph.2025.1608796 (PMC12722918; doi:10.3389/ijph.2025.1608796)
Supplement: Supplementary file 1 [file DataSheet1.docx]

**Supplementary File 1.** Differences in mental health and functional outcomes at baseline and 6-month follow-up (means and standard deviations). (Canary, Islands, Spain, 2024)

|  | **Baseline** | | | **6-month follow-up** | | | |
| --- | --- | --- | --- | --- | --- | --- | --- |
|  | **Control Group (VR)**  **(n = 32)** | **Intervention Group (IPS)**  **(n = 31)** | **p-value** | **Control Group (VR)**  **(n=19)** | **Intervention Group (IPS)**  **(n=24)** | **p-value** |  |
| **GAF** | 59.25 (9.23) | 61.26 (12.56) | 0.471 | 57.74 (8.33) | 63.83 (10.75) | 0.049 |  |
| **Quality of life** | 5.06 (0.84) | 5.16 (0.78) | 0.630 | 4.89 (0.94) | 5.21 (0.83) | 0.253 |  |
| **PANSS Positive** | 14.61 (4.66) | 14.42 (7.01) | 0.924 | 15.16 (5.08) | 14.88 (7.32) | 0.887 |  |
| **PANSS Negative** | 17.72 (5.57) | 18.13 (7.54) | 0.806 | 18.74 (6.37) | 19.38 (7.94) | 0.777 |  |
| **PANSS General** | 34.78(10.41) | 34.23 (11.66) | 0.842 | 37.79 (11.11) | 36.04 (12.32) | 0.632 |  |
| **HADS -Anxiety** | 4.56 (3.43) | 4.16 (3.57) | 0.651 | 5.0 (3.68) | 4.46 (3.73) | 0.637 |  |
| **HADS Depression** | 3.50 (3.21) | 4.00 (3.26) | 0.542 | 4.32 (3.43) | 4.33 (3.49) | 0.987 |  |
| **HADS-Global** | 8.06 (5.98) | 8.16 (6.10) | 0.948 | 9.32 (6.39) | 8.79 (6.59) | 0.794 |  |
| **GSDS Total** | 10.22 (3.99) | 9.39 (4.23) | 0.425 | 10.32 (4.08) | 9.17 (4.64) | 0.400 |  |
| **Academic self-concept** | 7.29 (1.41) | 7.68 (1.71) | 0.328 | 7.10 (1.31) | 7.39 (1.78) | 0.561 |  |
| **Social self-concept** | 6.43 (1.67) | 6.75 (1.94) | 0.482 | 6.51 (1.91) | 6.46 (2.10) | 0.931 |  |
| **Emotional self-concept** | 6.19 (2.37) | 6.61 (2.38) | 0.484 | 5.95 (2.45) | 6.11 (2.47) | 0.834 |  |
| **Family self-concept** | 7.74 (1.66) | 7.68 (2.10) | 0.899 | 7.63 (1.76) | 7.37 (2.21) | 0.682 |  |
| **Physical self-concept** | 5.51 (2.13) | 5.70 (2.42) | 0.741 | 10.32 (4.08) | 5.57 (2.46) | 0.783 |  |

VR: Vocational rehabilitation (train-then-place); IPS: Individual Placement and Support.

**Supplementary File 2.** Evolution of mental health and functional outcomes from baseline to 6-month follow-up in the IPS (blue line) and VR (red line) groups. (Canary, Islands, Spain, 2024)


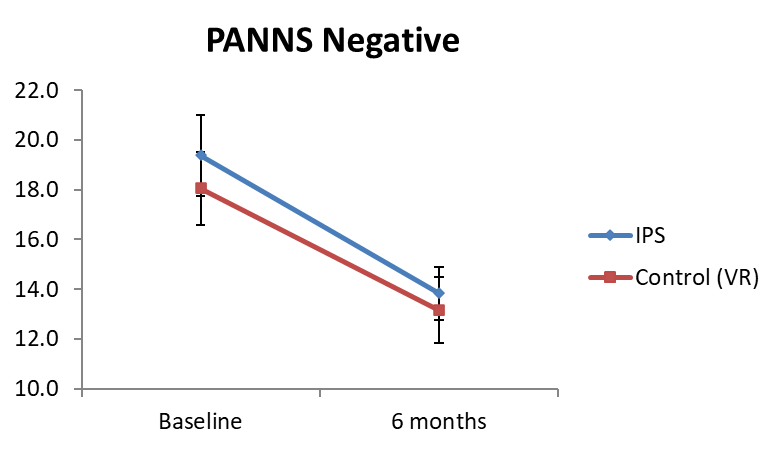

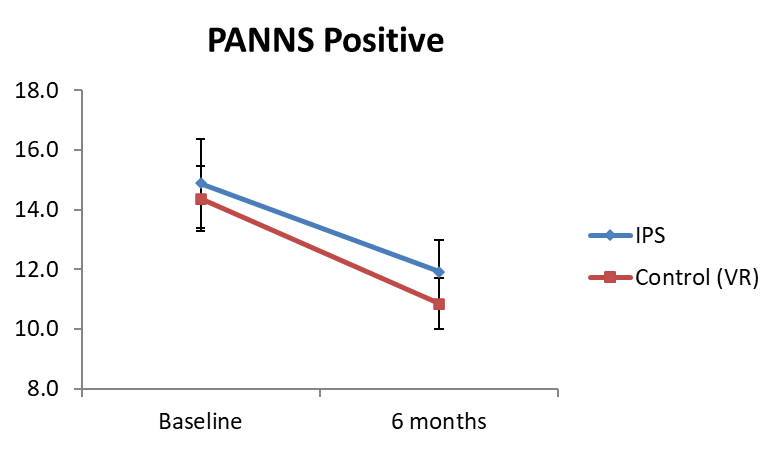

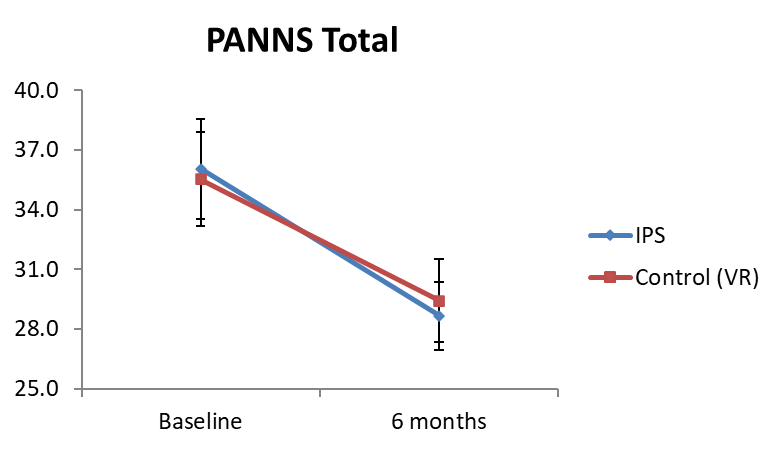

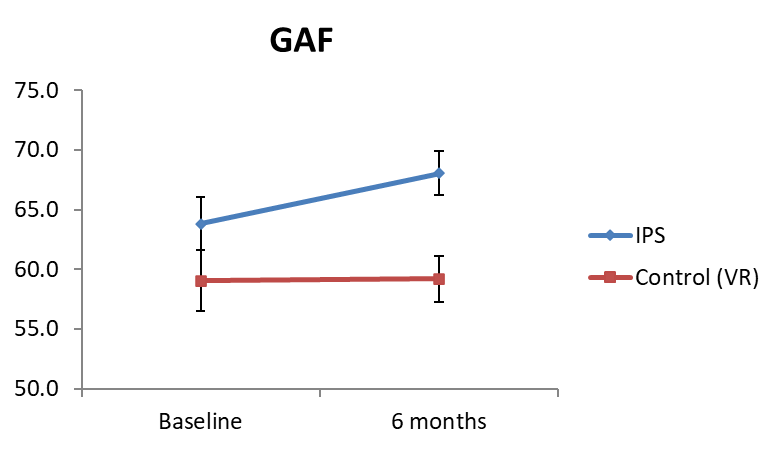

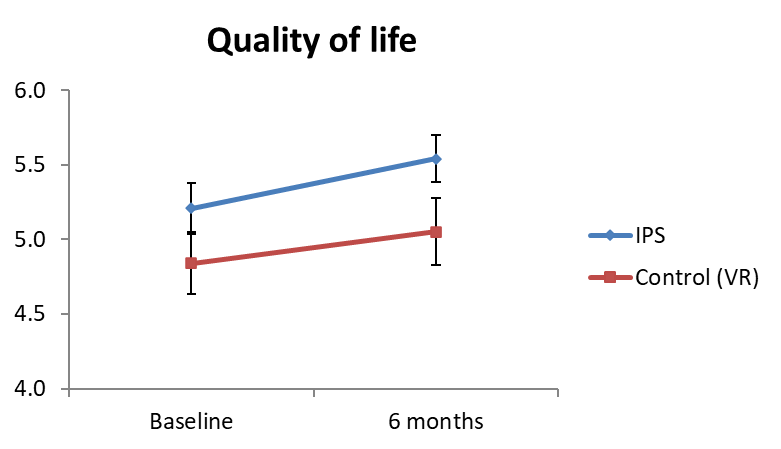

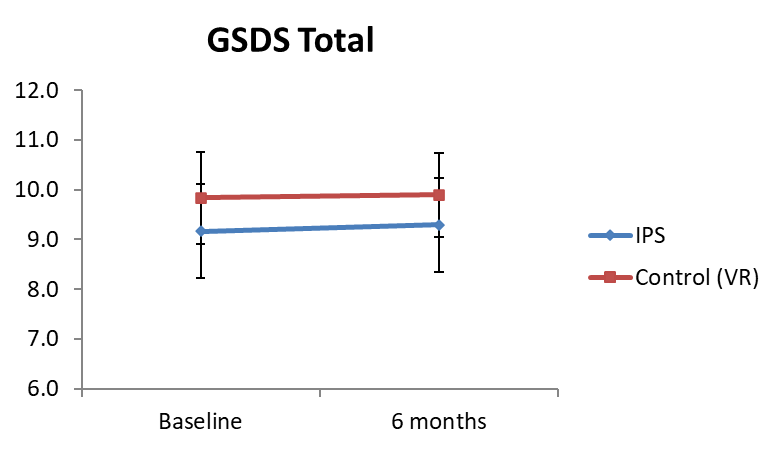

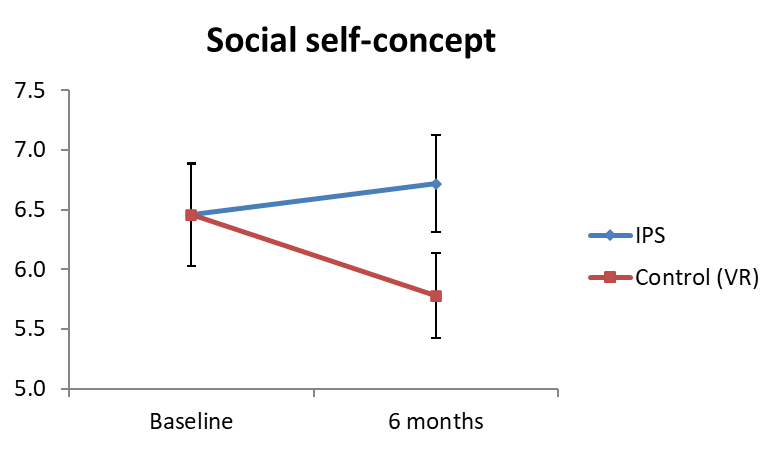

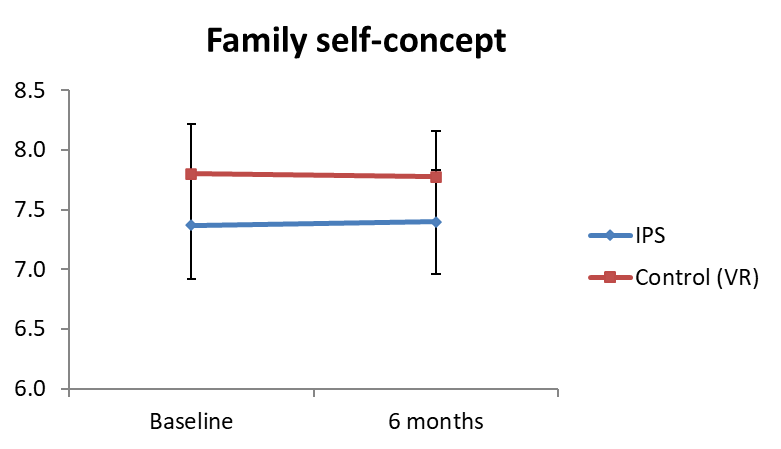

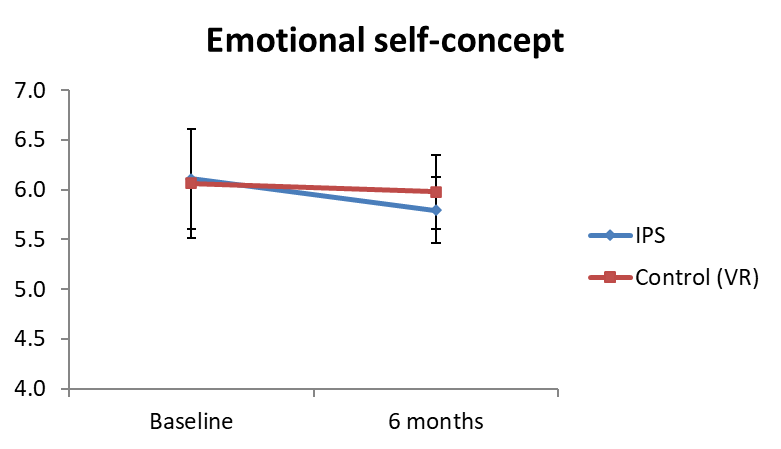

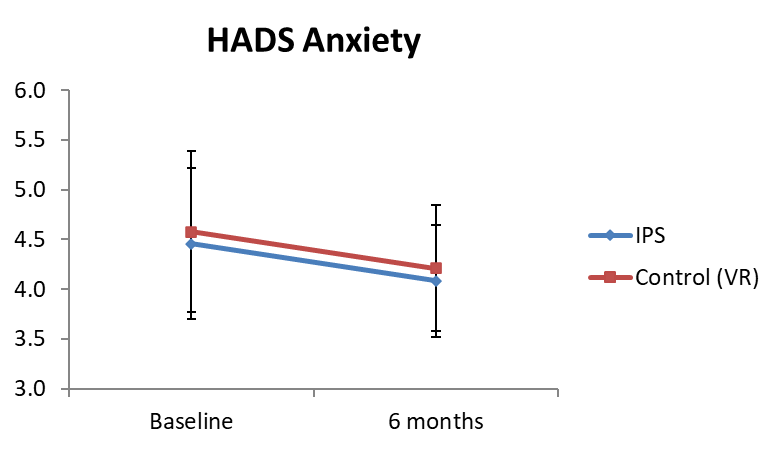

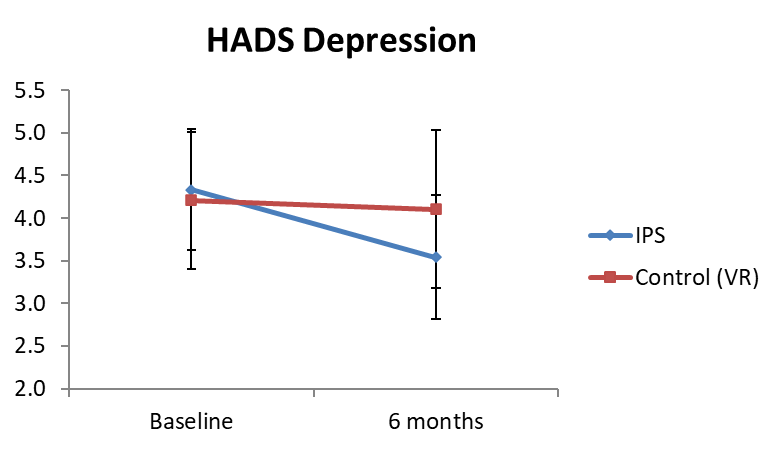

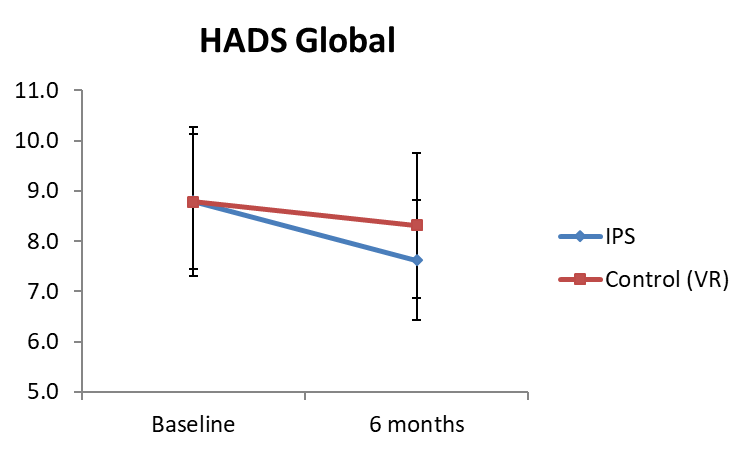


**Supplementary File 3.** Effect sizes (Cohen’s d and 95% confidence interval) for within-group and between-group changes in mental and functional outcomes from baseline to 6-month follow-up. (Canary, Islands, Spain, 2024)

|  | | **Within-group change*** | **Overall between-group difference†** | **Between-group difference over time‡** |
| --- | --- | --- | --- | --- |
| **Quality of life** | | -0.371 (-0.678; -0.060) | 0.547 (-0.070; 1.157) | 0.166 (-0.438; 0.768) |
| **GAF** | | -0.380 (-0.666; -0.059) | 0.728 (0.102; 1.346) | 0.646 (0.025; 1.260) |
| **PANSS** | Positive | 0.836 (0.484; 1.180) | 0.153 (-0.451; 0.754) | 0.147 (-0.457; 0.748) |
|  | Negative | 0.878 (0.522; 1.227) | 0.176 (-0.428; 0.778) | -0.107 (-0.709; 0.496) |
|  | General | 0.858 (0.504; 1.205) | -0.013 (-0.615; 0.589) | -0.158 (-0.760; 0.445) |
| **HADS** | Anxiety | 0.129 (-0.172; 0.428) | -0.043 (-0.645;0.559) | -0.002 (-0.604; 0.600) |
|  | Depression | 0.185 (-0.118; 0.485) | -0.065 (-0.667; 0.537) | -0.259 (-0.862; 0.347) |
|  | Global | 0.180 (-0.122; 0.481) | -0.059 (-0.661; 0.543) | -0.144 (-0.746; 0.460) |
| **Self-concept** | Academic | 0.292 (-0.015; 0.596) | 0.262 (-0.344; 0.865) | -0.033 (-0.635; 0.569) |
|  | Social | 0.116 (-0.185; 0.415) | 0.259 (-0.347; 0.862) | 0.743 (0.116; 1.361) |
|  | Emotional | 0.102 (-0.199; 0.401) | -0.040 (-0.641; 0.563) | -0.108 (-0.710; 0.495) |
|  | Family | -0.005 (-0.304; 0.294) | -0.210 (-0.812; 0.395 | 0.052 (-0.551; 0.653) |
|  | Physicist | 0.231 (-0.073; 0.532) | 0.216 (-0.388; 0.819) | 0.343 (-0.265; 0.947) |
| **GSDS Total** | | -0.060 (-0.359; 0.240) | -0.151 (-0.752; 0.453) | -0.460 (-0.556; 0.648) |

A negative value indicates:

* a higher score at six months compared to baseline.

† a higher score for the control group compared to the IPS group.

‡ that the change in score (from six months to baseline) was greater in the control group than in the IPS group.

**Supplementary File 4.** Evaluation of IPS/RT strategies on mental health and functional outcomes among completers and lost to follow-up participants (means and standard deviations). (Canary, Islands, Spain, 2024)

|  |  | **Completers**  **(n = 43)** | **Lost to follow-up**  **(n = 20)** | **p-value*** |
| --- | --- | --- | --- | --- |
| **Quality of life** | | 5.07 (0.88) | 5.20 (0.62) | 0.555 |
| **GAF** | | 61.14 (10.12) | 58.30 (12.60) | 0.342 |
| **PANSS** | **Positive** | 15 (6.36) | 13.40 (4.79) | 0.322 |
|  | **Negative** | 19.09 (7.21) | 15.40 (3.98) | 0.011 |
|  | **General** | 36.81 (11.69) | 29.55(7.18) | 0.013 |
| **HADS** | **Anxiety** | 4.70 (3.67) | 3.65 (2.96) | 0.269 |
|  | **Depression** | 4.33 (3.42) | 2.50 (2.35) | 0.035 |
|  | **Global** | 9.02 (6.43) | 6.15 (4.46) | 0.045 |
| **Self-concept** | **Academic** | 7.26 (1.57) | 7.96 (1.46) | 0.099 |
|  | **Social** | 6.48 (1.99) | 6.81 (1.32) | 0.443 |
|  | **Emotional** | 6.04 (2.43) | 7.17 (2.06) | 0.077 |
|  | **Family** | 7.48 (2.00) | 8.20 (1.48) | 0.156 |
|  | **Physicist** | 5.47 (2.48) | 5.83 (1.73) | 0.513 |
| **GSDS Total** | | 9.67 (4.39) | 10.10 (3.48) | 0.705 |

**
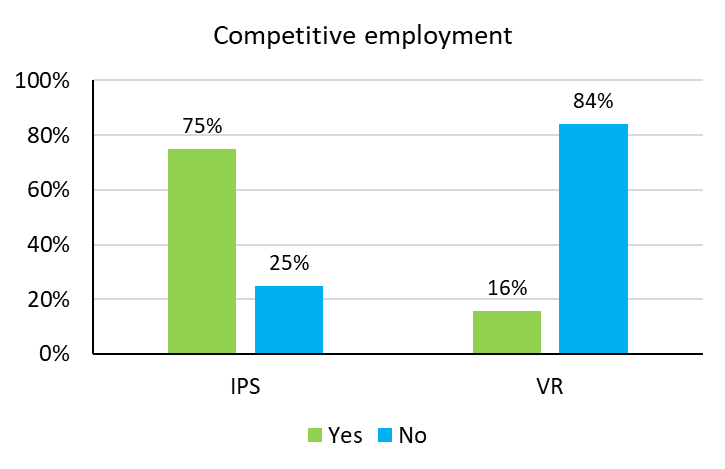
Supplementary File 5.** Proportion of participants achieving competitive employment in IPS and VR. (Canary, Islands, Spain, 2024)
